# Plasma cell and neutrophil enriched neovascularization with granulomatous lymphangitis in POEMS syndrome

**DOI:** 10.1016/j.jdcr.2024.03.025

**Published:** 2024-04-27

**Authors:** Oluwaseyi Adeuyan, Cynthia M. Magro, Megan H. Trager, Emily R. Gordon, Brigit A. Lapolla, Celine M. Schreidah, Lauren M. Fahmy, Larisa J. Geskin

**Affiliations:** a Columbia University Vagelos College of Physicians and Surgeons, New York, New York; b Department of Pathology and Laboratory Medicine, Weill Cornell Medicine, New York, New York; c Department of Dermatology, Columbia University Irving Medical Center, New York, New York

**Keywords:** cutaneous T-cell lymphoma, kappa light chain restriction, POEMS syndrome

## Introduction

Polyneuropathy, organomegaly, endocrinopathy, monoclonal plasma cell disorder and skin changes (POEMS) syndrome is a rare paraneoplastic disorder associated with plasma cell malignancies that is frequently lambda light chain restricted.^1^ POEMS syndrome is distinguished from other paraproteinaemic neuropathies by its multiorgan involvement which may be partly explained by the increased secretion of angiogenic and proinflammatory cytokines.^1^ We present a unique case of kappa restricted POEMS syndrome with atypical clinicohistopathologic features in a 75-year-old man.

## Case report

A 75-year-old man with personal history of anemia, fatigue, bilateral hand numbness and difficulty walking with transient “rashes” and a family history of cutaneous T-cell lymphoma (CTCL) in his son presented to his local dermatologist with an asymptomatic “growth” on the right buttock 1 month following hip arthroplasty. He was taking azathioprine 2 months prior to initial presentation given concern for rheumatologic disease although autoimmune workup over the past 10 years remained inconclusive.

A biopsy of the “growth” was performed by his local dermatologist with findings concerning for CTCL given a CD4 and CD30 positive chronic inflammatory infiltrate with positive clonal T-cell receptor beta gene rearrangement.

**Fig 1:**
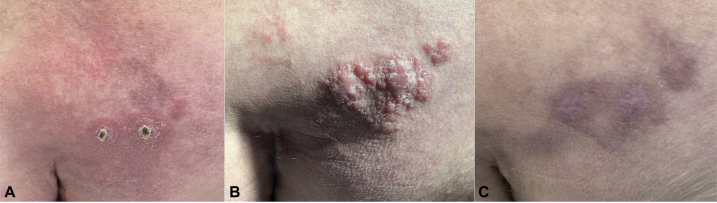
**A,** Erythematous, smooth plaque with 2 healing biopsy sites of right medial buttock at initial visit. **B,** The plaque evolved into a firm pink tumor 4 weeks after initial visit. **C,** Resolution of skin findings with mild hyperpigmentation on the right buttock 5 months after initial visit.

Upon presentation to our institution 3 weeks later, he was cachectic with an unbalanced gait. Physical exam revealed an erythematous smooth plaque with 2 healing biopsy sites on the right buttock (Fig 1). Blood work as part of the evaluation for CTCL included complete blood count, complete metabolic profile, lactate dehydrogenase, beta-2-microglobulin and flow cytometry, which were all normal. At the 1-month follow-up visit, the initial plaque had progressed into a firm pink tumor (Fig 1). Additional findings included leukonychia with mild acrocyanosis and peripheral edema.

**Fig 2:**
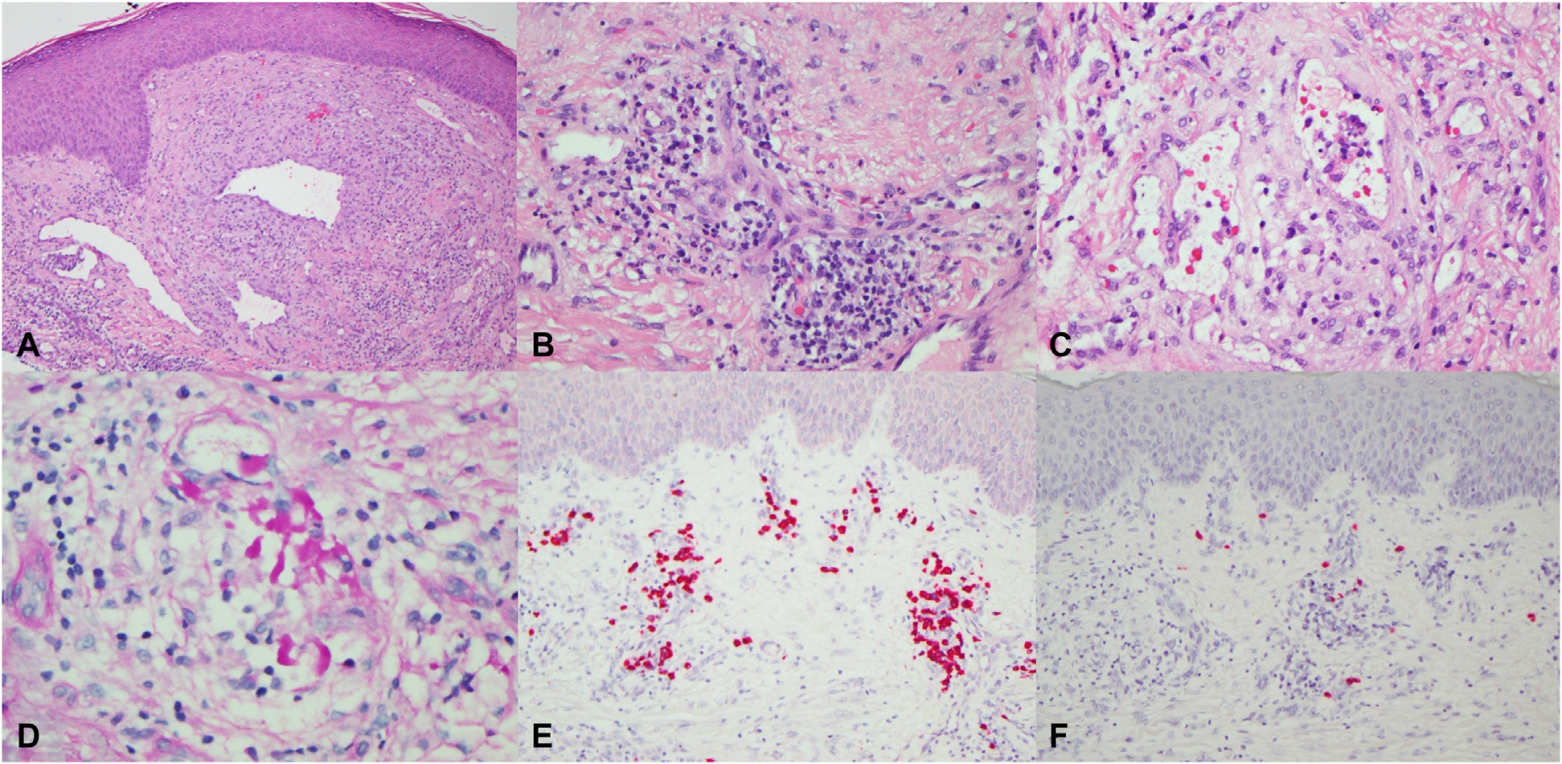
**A,** The biopsy showed a very unusual combined picture of neovascularization and inflammation. The vascular structures are clearly the epicenter of the supervening striking inflammatory process prevalent throughout the sample. The vessels range from congeries of capillaries and venules to markedly dilated, thin-walled structures of lymphatic derivation. In this particular image there are irregular dilated vessels exhibiting a somewhat sinusoidal growth pattern (H&E, 200×). **B,** The vascular proliferations are associated with a mixed infiltrate of histiocytes, neutrophils, many plasma cells, and lymphocytes. (H&E, 400×). **C,** In certain foci, there is occlusion of vascular structures by many histiocytes whereby the degree of intravascular histiocytic infiltration defines a component of an occlusive granulomatous lymphangitis. (H&E, 400×). **D,** Some vessels demonstrate intravascular deposits of proteinaceous material consistent with immunoglobulin. The PASD stains highlight the abnormal proteinaceous precipitates within the vascular lumens. (PASD, 400×). **E,** The kappa preparation highlights the plasma cells very extensively. (Kappa ISH stain, 200×). **F,** In contradistinction, the degree of immunoreactivity for lambda is relatively minimal. (Lambda ISH stain, 200×). *H&E*, Hematoxylin and eosin; *ISH*, in situ hybridization; *PASD*, periodic acid-Schiff with diastase.

A review of outside slides, which had been previously interpreted as a T cell dyscrasia, disclosed a very striking mixed inflammatory cell infiltrate exhibiting accentuation around congeries of proliferating blood vessels and dilated lymphatics (Fig 2, *A*). The infiltrate was comprised of neutrophils, lymphocytes, histiocytes, and many plasma cells (Fig 2, *B*). In addition, there was a component of granulomatous lymphangitis characterized by intravascular collections of histiocytes, some of which contained engulfed immunoglobulin (Fig 2, *C* and *D*). The plasma cells exhibited kappa light chain restriction (Fig 2, *E*). Thus, a pathological diagnosis was rendered by CMM of a kappa light chain restricted plasma cell dyscrasia within a background of neovascularization and granulomatous lymphangitis highly suspicious for AESOP/POEMS syndrome or multicentric Castleman’s disease. A repeat biopsy confirmed the findings on the earlier biopsy; an Ig kappa light chain assay by polymerase chain reaction disclosed monoclonality.

Given the concern for an underlying plasma cell dyscrasia, serum protein electrophoresis, urine protein electrophoresis, vascular endothelial growth factor (VEGF) with interleukin (IL)-6 serum levels and endocrine studies were performed. Blood work revealed significant kappa chain predominance with kappa/lambda ratio elevated to 2.35, chronic anemia and lymphopenia. His blood free kappa chain was elevated, and serum protein electrophoresis was notable for chronic inflammatory pattern but no M spike. VEGF was elevated to 96 pg/mL (reference range: 31-86 pg/mL) and IL-6 was significantly elevated to 54 pg/mL (reference: <5 pg/mL). Endocrine workup was unremarkable.

Fat pad biopsy was performed to rule out light chain amyloidosis, which was negative. Whole body positron emission tomography-computed tomography was obtained given suspicion for AESOP or POEMS syndrome which revealed multiple hypermetabolic retroperitoneal and pelvic lymphadenopathy, where plasmacytoma could not be excluded. Subsequent CT guided biopsy of the right para-caval lymph node was negative. Bone marrow biopsy was performed to assess marrow involvement by a clonal population, which showed mild plasmacytosis. To evaluate polyneuropathy, electromyography and nerve conduction studies were performed, which showed electrophysiologic evidence of a mild sensorimotor neuropathy, severe bilateral median mononeuropathies, and chronic radiculopathy but no demyelinating polyneuropathy.

The patient was therefore diagnosed with POEMS syndrome given his polyneuropathy, monoclonality, VEGF elevation, lymphadenopathy, and skin changes. Azathioprine was discontinued given minimal clinical improvement. Localized radiation to the right buttock was recommended. Although his skin findings largely regressed spontaneously without treatment within 6 months (Fig 1), he continued to experience severe neuropathy without progression and has been participating in physical and occupational therapy.

## Discussion

We present an atypical clinical presentation with unusual histopathologic findings of kappa light chain restricted POEMS syndrome. The dermatopathology findings were unique given the combined lymphatic and blood vessel neovascularization with neutrophilic and plasmacytic infiltrates and the presence of granulomatous lymphangitis with localization of immunoglobulin within intravascular histiocytes. The neovascularization was likely a paraneoplastic VEGF effect while the significant neutrophilic infiltrates could be secondary to IL-6 production by neoplastic plasma cells. We are not aware of prior reports of POEMS syndrome describing reactive/paraneoplastic granulomatous lymphangitis. This rare but distinctive reaction pattern is most commonly associated with sarcoidosis, Crohn’s disease and orofacial granulomatosis.^2^

POEMS syndrome represents a multisystemic paraneoplastic process secondary to an underlying plasma cell neoplasm.^3^ Patients with POEMS syndrome must fulfill both mandatory criteria with at least 1 of 3 major criteria and 1 of 6 minor criteria (Table I).^3^ Aside from the lack of an underlying plasmacytoma, the patient would have met criteria for AESOP syndrome. Thus, a modified variation of AESOP syndrome which we term here as “AESUP” (adenopathy and extensive skin patch *underlying a plasma cell dyscrasia*) syndrome may have future diagnostic utility.

**Table I:** Criteria for the diagnosis of plasma cell disorder and skin changes syndrome,^3^ including the criteria that this case fulfills

Criteria		Present case
Mandatory	Polyneuropathy (typically demyelinating)	+ (sensorimotor polyneuropathy, chronic radiculopathy)
	Monoclonal plasma cell-proliferative disorder (>95% cases with lambda-restriction)	+ (kappa-restricted)
Major	Castleman disease	-
	Sclerotic bone lesions	-
	VEGF elevation	+ (mild)
Minor	Organomegaly (splenomegaly, lymphadenopathy, or hepatomegaly)	+ (lymphadenopathy)
	Extravascular volume overload (edema, pleural effusion, or ascites)	+ (edema)
	Endocrinopathy (adrenal, thyroid, pituitary, gonadal, parathyroid, pancreatic)	-
	Skin changes (hyperpigmentation, hypertrichosis, glomeruloid hemangiomata, plethora, acrocyanosis, flushing, leukonychia)	+ (erythematous plaque → firm tumor, mild acrocyanosis, leukonychia)
	Papilledema	
	Thrombocytosis/polycythemia	

*VEGF*, Vascular endothelial growth factor.

POEMS syndrome is overwhelmingly (ie, >90% of cases) lambda chain restricted, although isolated cases of kappa restriction or bi-clonal gammopathy have been described.^1^^,^^4^ These isolated cases have demonstrated either demyelinating polyneuropathy alone or with axonal degeneration, except for 1 case in which the neuropathy was not characterized.^4^, ^5^, ^6^, ^7^ However, there was no evidence of demyelinating polyneuropathy in this case. Additionally, all previously reported kappa restricted cases showed evidence of endocrinopathy, which is absent here.^5^ Thus, this patient demonstrated a new, unreported constellation of signs (Table I). Although the role of kappa versus lambda restriction is unknown in POEMS syndrome, there are data suggesting that patients with kappa restricted monoclonal gammopathy of unknown significance chiefly present with axonal type neuropathy compared to their lambda restricted counterparts, an observation not present in this case.^8^

The pathogenesis of POEMS syndrome remains unclear. Studies have proposed that malignant clonal plasma cells may drive lymphoid stroma proliferation and IL-6 production, which promotes abnormal secretion of VEGF. The consequent procoagulant state drives hypoxia induced factor alpha-1 production which further drives VEGF production and release of pro-inflammatory cytokines.^9^ VEGF levels may serve as a prognostic biomarker for POEMS syndrome.^1^^,^^9^ However, anti-VEGF therapy may not lead to clinical improvement.^9^^,^^10^ One hypothesis suggests that suppression of VEGF alone is insufficient to inhibit disease activity since multiple cytokines are involved.^10^ Moreover, sudden withdrawal of VEGF, an important factor for blood vessel development, may lead to apoptosis in endothelial cells, resulting in increased capillary leakage and temporary clinical decline.^10^

In POEMS syndrome, systemic and skin findings tend to resolve before the neuropathy.^11^ Radiation therapy has excellent prognosis in patients with limited disease. For patients with neuropathy, supportive measures such as physical therapy may be beneficial.^11^

In conclusion, we present an unusual case of POEMS syndrome. The atypical hybrid lymphatic blood vessel neovascularization with overlapping granulomatous lymphangitis on histology represents cytokine sequelae of a plasma cell driven process. This case demonstrates the importance of maintaining clinical suspicion for POEMS syndrome when presented with uncommon features.

## Conflicts of interest

LJG has served as an investigator for and/or received research support from Helsinn Group, J&J, Mallinckrodt, Kyowa Kirin, Soligenix, Innate, Merck, BMS, and Stratpharma; on the speakers’ bureau for Helsinn Group and J&J; and on the scientific advisory board for Helsinn Group, J&J, Mallinckrodt, Sanofi, Regeneron, and Kyowa Kirin. OA, CMM, MHT, ERG, BAL, CMS and LMF have no conflicts of interest to declare.
